# Reinforced locking screws with enhanced head and neck junction: improving biomechanical stability in opening-wedge high tibial osteotomy applications

**DOI:** 10.1186/s13018-025-06015-4

**Published:** 2025-06-19

**Authors:** Kyung-Wook Nha, Hyungsuh Kim, Kyoung-Tak Kang, Jae Gyoon Kim, Hyung Jun Park

**Affiliations:** 1 Department of Orthopedic Surgery, Ilsanpaik Hospital, Inje University College of Medicine, Goyangsi, Republic of Korea; 2 Skyve R&D Lab, Seoul, Republic of Korea; 3 Department of Mechanical Engineering, Yonsei University, Seoul, Republic of Korea; 4 Department of Orthopedic Surgery, Korea University Ansan Hospital, Korea University College of Medicine, 123, Jeokgeum-ro, Danwon-Gu, Ansan-si, 425-707 Gyeongki-do Republic of Korea

**Keywords:** Reinforced locking screws, Biomechanical stability, Head and neck junction, Opening-wedge high tibial osteotomy, Screw breakage prevention

## Abstract

**Background:**

Locking plates and screws are essential in orthopedic surgeries, including opening-wedge high tibial osteotomy (OWHTO), owing to their ability to provide angular stability and support under mechanical stress. However, screw breakage at the head and neck junction remains a significant issue, compromising fixation and requiring revision surgery. This study aimed to determine whether increasing the diameter of the locking screws at the head and neck junctions enhances biomechanical stability.

**Methods:**

Sixty screws, divided into two groups (*n* = 30 per group) —a non-reinforced group with a standard design and a reinforced group with an increased head and neck diameter— were tested. Both groups consisted of 5.0 mm locking screws, each with a length of 80 mm. Biomechanical testing, which included compression, fatigue, and torsional strength tests, was conducted to reflect clinically relevant conditions.

**Results:**

The reinforced screws demonstrated significantly superior biomechanical performance. In the compression test, they exhibited a higher load to failure (909.0 ± 30.4 N vs. 757.5 ± 46.2 N, *p* < 0.001). In the fatigue test, the reinforced screws endured more cycles before failure (70788.6 ± 6310.6 cycles vs. 23016.2 ± 5,157.9 cycles, *p* < 0.001) and had a greater displacement distance (3.0 ± 0.4 mm vs. 2.3 ± 0.3 mm, *p* = 0.001). The torsional test showed higher torque at failure for the reinforced screws (17.3 ± 0.3 Nm vs. 16.5 ± 0.4 Nm, *p* < 0.001), although the angular displacement differences were not statistically significant (202.0° ± 63.9° vs. 247.2° ± 64.9°, *p* = 0.105).

**Conclusions:**

Reinforcing the head and neck junction of locking screws significantly improves their biomechanical performance. These findings suggest that structural modifications can reduce hardware failure risks in high-stress procedures such as OWHTO, enhancing implant durability and clinical outcomes.

## Background

Locking plates and screws are advanced biomechanical devices designed to stabilize fracture sites and provide structural integrity during the bone-healing process [1–3]. These devices offer angular stability and can be applied using minimally invasive techniques [1–3]. This makes them suitable not only for patients with complex fractures and osteoporosis but also for stabilizing osteotomy sites after lower extremity realignment surgeries, such as opening-wedge high tibial osteotomy (OWHTO) [2–5]. 

**Fig. 1:**
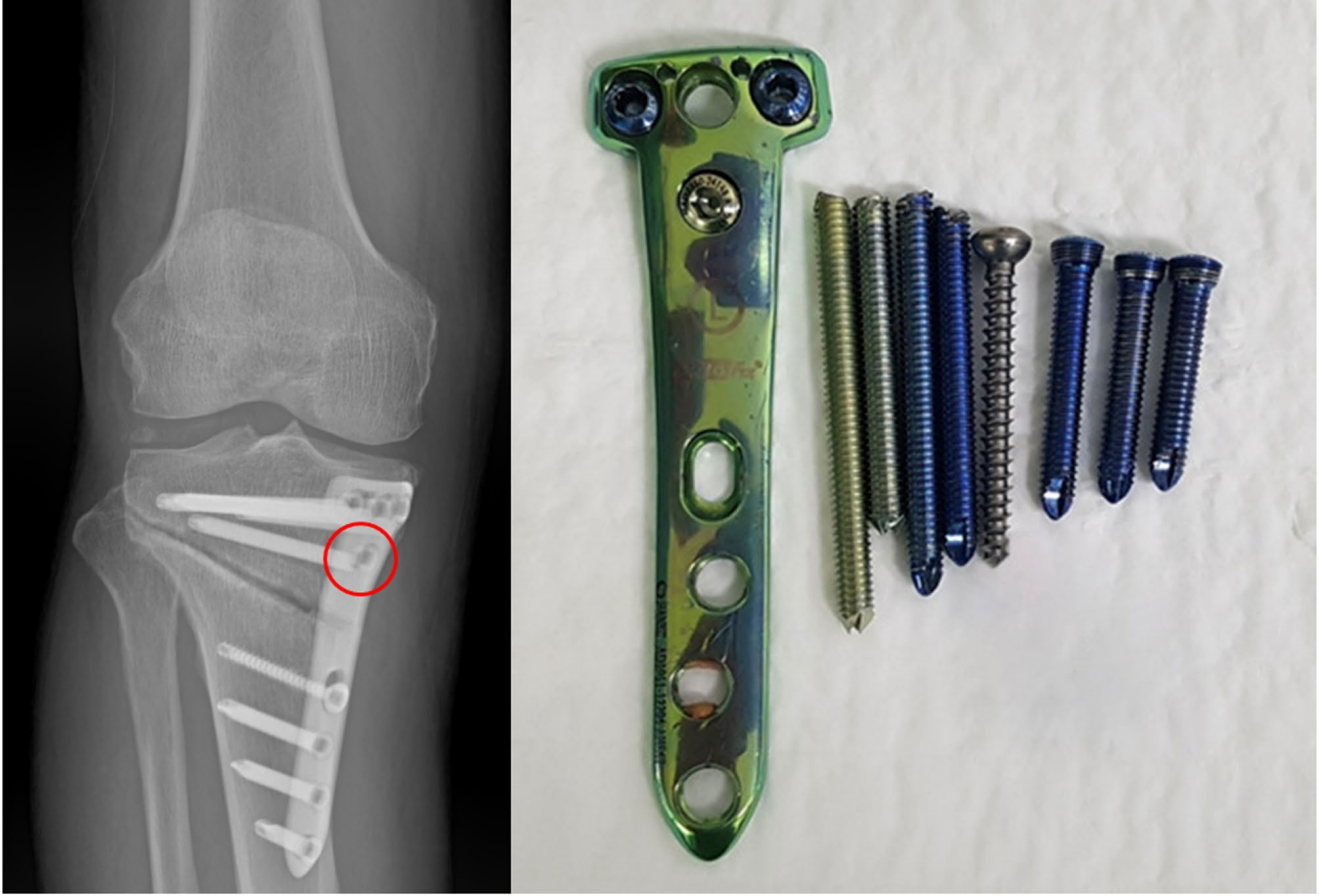
Locking screw breakage at the head and neck junction in opening-wedge high tibial osteotomy. Postoperative radiograph showing fixation with locking plate and screw. The red circle indicates definite screw breakage. Removed hardware components

Despite their advantages, locking plates and screws have several technical disadvantages and complications [1, 6, 7]. One common issue is the stripping of screw heads, which makes their removal challenging [1]. Additionally, screw breakage at the junction of the head and neck regions has been reported [6, 8]. These failures are not limited to fracture-related surgeries but have also been reported in OWHTO [6–10]. Such incidents compromise fixation strength, increase the risk of non-union, and may necessitate revision surgery, posing significant clinical concerns [11, 12]. Especially, in OWHTO, a loss of reduction may occur, compromising the surgical objective of realignment intended to alleviate loading on the medial compartment of the knee joint [9, 13]. Therefore, surgeons and manufacturers have focused on developing optimal fixation strategies or implementing structural reinforcements to address these challenges [12–15]. Previous studies of OWHTO have reported adjusting the screw length or altering the screw types to preserve fixation strength while promoting gap healing [13–15]. Despite these efforts, locking screw breakage has occasionally been observed after OWHTO (Fig. 1). Such breakages predominantly occur at the junction of the head and neck region, which is recognized as the weakest point of the screw [16, 17] However, it is well established that increasing the screw thickness enhances its mechanical strength [18, 19]. Considering these principles, increasing the diameter at the head and neck junction may improve mechanical stability and reduce the likelihood of structural failure [18, 19] However, previous studies have not investigated biomechanical modifications aimed at reinforcing the junction of the head and neck region to mitigate screw breakage, especially in lower-extremity realignment surgeries.

We aimed to investigate whether increasing the diameter of the locking screw at the junction of the head and neck region enhances their biomechanical stability. We hypothesized that expanding the diameter of the locking screw would improve biomechanical performance.

## Method

### Study design

**Fig. 2:**
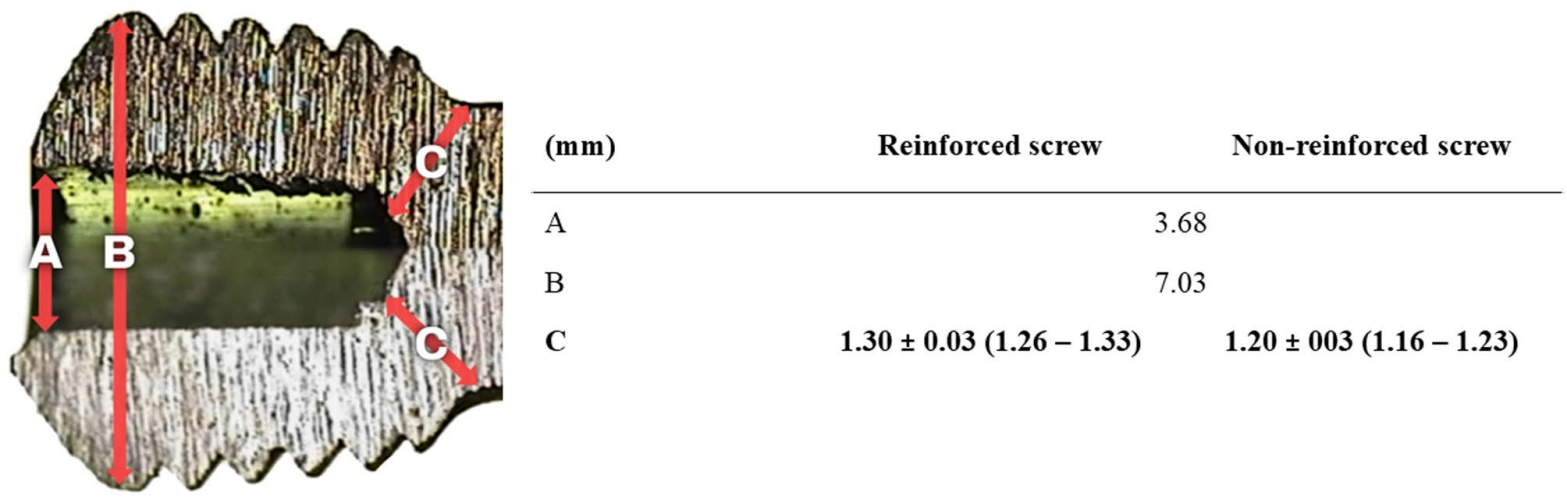
Structural differences between reinforced and non-reinforced screws. Distance across the space where the screwdriver engages within the screw head. Outer diameter at the widest point of the screw head. Width of the screw head and neck junction.Values are presented as mean ± standard deviation

**Fig. 3:**
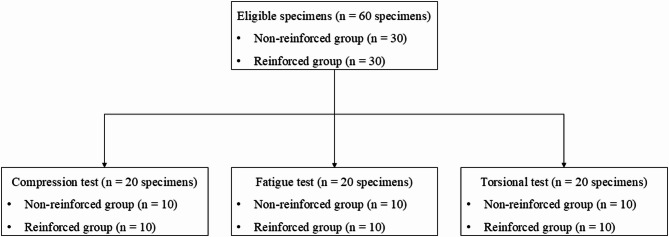
Flowchart of screw allocation for biomechanical testing

This study included 60 screws, divided into two groups of 30 screws each. The screws were categorized as non-reinforced or reinforced based on structural modifications at the head and neck junctions. Both groups consisted of 5.0 mm locking screws with a length of 80 mm (the longest screw length available). The non-reinforced group consisted of standard locking screws. In contrast, the reinforced group included screws with an increased diameter at the head and neck junction, with a mean range of 1.20 to 1.30 mm (Fig. 2), without any other structural changes. To accurately evaluate the dimensions of specific screw regions, two screws were randomly selected from each group, manually split, and measured using direct caliper measurements (ATOS Q; Carl Zeiss GOM Metrology GmbH; Braunschweig, Germany). Each screw was randomly assigned a number and allocated to one of the two groups using blocked randomization performed using Microsoft Office Excel 2021 (Microsoft Corporation, Redmond, WA, USA). Thirty screws from each group were selected for biomechanical testing (Fig. 3).

### Biomechanical testing

Biomechanical testing was performed to evaluate screw stability under clinically relevant conditions. The assessment included tests of three parameters: compression, fatigue, and torsional strength. Compression and fatigue tests were designed to replicate the repetitive and compressive loads encountered during postoperative activities using a universal tensile and compression testing machine (DUT 2000 C; Dae Kyung Tech, Gumi, South Korea) and a fatigue tester (DTF-505 model, Dae Kyung Tech). A torsional strength test was used to evaluate the resistance of the screws to rotational forces during fixation and removal procedures, simulating the mechanical stresses encountered in clinical practice. A torque tester (DT&T/DTF-T100; Dae Kyung Tech) was used for the torsional strength tests.

A compression strength test was conducted to determine the maximum load that the screws could withstand before structural failure. To perform the test, locking plates and screws were assembled and securely fixed. A compressive force was applied to the mid-portion of the screw through an upper jig at a speed of 10 mm/min until structural failure occurred, and the load at failure was measured. Fatigue strength tests were performed to evaluate the durability of the screws under cyclic loading conditions. The locking plates and screws were assembled and fixed, after which cyclic loads ranging from 12 to 120 N were applied at a frequency of 5 Hz. The test continued until screw failure occurred, and both the number of cycles and the total displacement distance of the screw tip until failure was recorded. Finally, the torsional strength test involved applying a rotational force to the screw at a speed of 1 rpm, after the locking plates and screws were assembled. The torque and angular displacement at the point of failure were measured.

### Statistical analysis

The Mann-Whitney U test was used to compare the mean values of the two groups. The sample size was calculated based on the three variables used to evaluate biomechanical stability: compression, fatigue, and torsional strength. The final sample size was determined by selecting the largest sample size required to achieve statistical significance among these variables. These calculations were performed using the results derived from the initial five experiments. For the torsional test, 16 samples (eight per group) were required to detect an effect size of d = 2.40, with an alpha level of 0.05 and a power of 0.99, using a two-sided test. Considering a 20% drop-out rate, 20 samples (10 per group) were necessary. Power analysis was conducted using G-Power version 3.1.9.7 (Franz Faul; Universität Kiel, Kiel, Germany). All statistical analyses were performed using SPSS version 20 (SPSS Inc., Chicago, IL, USA), with statistical significance set at a *p*-value < 0.05.

## Results

**Fig. 4:**
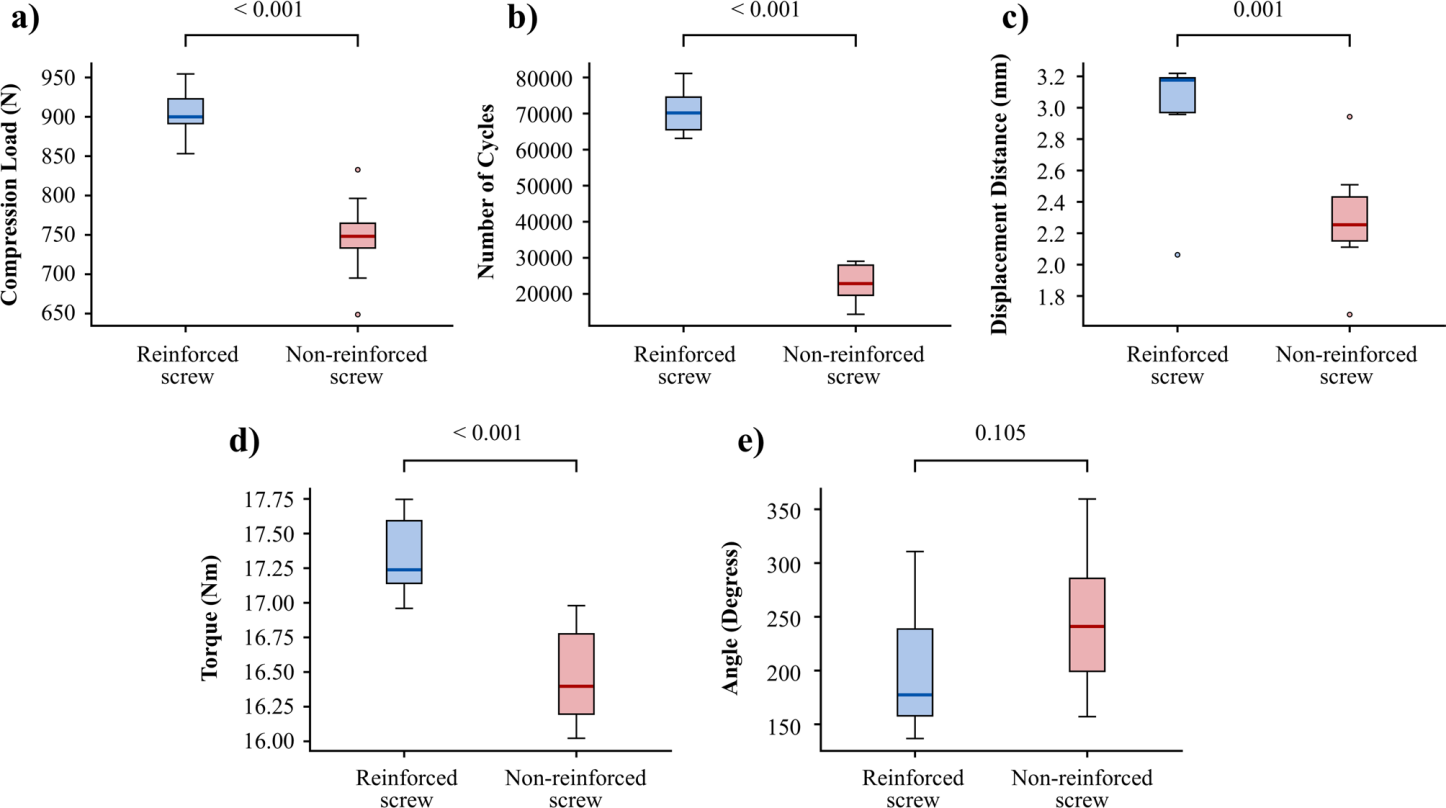
Biomechanical test results for reinforced and non-reinforced screws. Compression strength test: maximum load before failure. Fatigue strength test: number of cycles before failure. Fatigue strength test: displacement distance of the screw tip before failure. Torsional strength test: torque at failure. Torsional strength test: angular displacement at failure

Reinforced screws demonstrated superior stability compared with the non-reinforced screws. In the compression test, the reinforced screws exhibited a significantly higher load to failure than the non-reinforced screws, with mean values of 909.0 ± 30.4 N and 757.5 ± 46.2 N, respectively (*p* < 0.001, Table 1, Fig. 4a). In the fatigue test, the reinforced screws showed superior durability, enduring a significantly higher number of cycles before failure compared to the non-reinforced screws (70,788.6 ± 6,310.6 cycles and 23,016.2 ± 5,157.9 cycles, respectively, *p* < 0.001, Table 1, Fig. 4b). Additionally, the mean displacement distance was greater in the reinforced group than in the non-reinforced group (3.0 ± 0.4 mm and 2.3 ± 0.3 mm, respectively, *p* = 0.001, Table 1, Fig. 4c).

**Table 1:** Comparison of Biomechanical properties between non-reinforced and reinforced screws*

	Non-reinforced (*n* = 10)	Reinforced (*n* = 10)	*p*-value †
Compressive strength test			
Load to failure, N	757.5 ± 46.2	909.0 ± 30.4	< 0.001
Fatigue strength test			
Displacement distance, mm	2.3 ± 0.3	3.0 ± 0.4	0.001
Cycles to failure, n	23016.2 ± 5157.9	70788.6 ± 6310.6	< 0.001
Torsional strength test			
Torgue at failure, Nm	16.5 ± 0.4	17.3 ± 0.3	< 0.001
Angular displacement, °	247.2 ± 64.9	202.0 ± 63.9	0.105

* Values are presented as mean ± standard deviation

† Statistical analysis: Mann-Whitney U test

Abbreviation: N, newton; mm, millimeter; n, number; Nm, newton-meter

The reinforced screws demonstrated enhanced stability during torsional testing. The mean torque required for failure was significantly higher in the reinforced group, with a value of 17.3 ± 0.3 Nm, compared to 16.5 ± 0.4 Nm in the non-reinforced group (*p* < 0.001, Table 1, Fig. 4d). However, the mean angular displacement showed no statistically significant difference between the two groups, with values of 202.0 ± 63.9° for reinforced screws and 247.2 ± 64.9° for non-reinforced screws (*p* = 0.105, Table 1, Fig. 4e).

## Discussion

Locking plates and screws are widely used in orthopedic procedures owing to their ability to provide angular stability and support under mechanical stress [1–3]. However, mechanical failure, particularly at the head and neck junction of the screws, remains a significant concern, as it can compromise fixation and lead to revision surgeries [1, 6, 7]. Previous studies have analyzed risk factors and stress patterns associated with screw breakage but have largely overlooked direct structural modifications to enhance strength [6–9]. The principal finding of our study is that reinforcing the head and neck junction of locking screws significantly enhances their biomechanical performance, particularly in terms of durability under repetitive loading and torsional strength. This improvement underscores the potential of structural modifications to address the mechanical limitations of the current designs of locking screws used in orthopedic surgeries.

Our results confirmed the hypothesis that reinforcing the head and neck junctions of locking screws enhances their biomechanical stability. Several studies have reported breakage at these junctions, indicating a critical point of mechanical weakness [12, 13]. One study showed 28.6% of patients (12 out of 42) who underwent open reduction and internal fixation for distal femur fractures experienced proximal locking screw breakage [12]. The breakage predominantly occurred at the head and neck junctions. It was associated with specific risk factors, including advanced age, dominant limb usage, cortical screw application, and high plate-screw density. Locking screw breakage has also been documented as a complication after OWHTO [10]. A study evaluating clinical outcomes and complications after simultaneous bilateral OWHTO reported a case of delayed lateral hinge fracture and locking screw breakage [10]. The breakage exclusively occurred on the side without a metal block, which had been known to enhance fixation stability [20]. These findings suggest that initiating early weight-bearing rehabilitation under the condition of suboptimal fixation may increase stress on the head and neck junctions of locking screws, leading to failure [16, 17]. In addition to clinical studies, biomechanical investigations have provided insights into the mechanical behavior of locking screws in OWHTO [13]. A three-dimensional simulation study evaluated the effects of screw length and type on the mechanical stability of the locking plate and screw fixation [13]. Using an OWHTO model with a 12° opening, the study revealed that the length of the proximal screw had no significant impact on the stress distribution or osteotomy site gap changes. However, the screw type significantly influenced these factors. Far-cortical locking screws increased inter-fragmentary movement, nearly doubling gap deformation from 0.222 mm to 0.442 mm, thereby facilitating bone healing through enhanced micro-motion at the osteotomy site. However, this design also imposed a substantial mechanical burden on the screws, with stress levels rising approximately fourfold from 137.3 MPa to 541 MPa, increasing the likelihood of screw failure. Unlike previous studies that primarily examined risk factors and stress distribution, our study directly reinforced the head and neck junctions of locking screws to reduce the risk of mechanical failure. A previous study reported that increasing the diameter of locking screws by 20% resulted in a 20–25% improvement in fatigue strength [21]. Consistent with these findings, we found that increasing the diameter at the head and neck junction significantly improved both compressive and fatigue strength, confirming the superior biomechanical performance of reinforced screws. Additionally, the reinforced group exhibited a substantial increase in the number of cycles to failure in the fatigue test, indicating prolonged durability under cyclic loading conditions. Consequently, the displacement distance of the screw tip increased, likely because of the prolonged duration of stress application before failure. Therefore, our findings highlight the clinical significance of the structural reinforcement of locking screws at the head and neck junction as an effective strategy for enhancing biomechanical stability and reducing the risk of screw failure in orthopedic surgeries.

Our results confirmed the hypothesis that reinforcing the head and neck junctions of locking screws enhances torsional strength. Previous studies have established a relationship between screw diameter and mechanical stability [22–24]. One study evaluated the biomechanical stability of 2.7 mm and 3.5 mm locking compression plates (LCPs) using 21 composite ulnar specimens with oblique fracture lines [24]. The fractures were stabilized with either 2.7–3.5 mm LCPs, and the specimens were subjected to cyclic axial torsion and bending until failure. The study demonstrated that the 3.5 mm LCP was more effective in limiting fracture motion than the 2.7 mm LCP, with significantly reduced axial rotation in the 3.5 mm group (2°–3.5°) compared to the 2.7 mm group (5°–10°, *p* < 0.01). Similarly, another study used a large-volume bone loss model in sheep to evaluate the biomechanical performance of various internal fixation devices [22]. Their findings demonstrated that a 10 mm interlocking nail exhibited superior torsional and bending stiffness compared with an 8 mm nail, reinforcing that increased implant diameter contributed to enhanced mechanical stability. In our study, reinforced screws with an increased diameter exhibited significantly higher torsional strength than non-reinforced screws. This structural enhancement is expected to reduce the risk of locking screw breakage, which, although rare, can occur during hardware removal in orthopedic surgeries [7, 25]. Although there was no statistically significant difference in angular displacement at failure, the reinforced group showed a lower mean value (202.0° ± 63.9°) compared to the non-reinforced group (247.2° ± 64.9°). Although the difference did not reach statistical significance, the observed trend suggested that increased stiffness at the head and neck junction may contribute to reducing deformation under torsional loads [23]. Therefore, our findings emphasize the importance of structural modifications in optimizing the biomechanical performance of locking screws. Incorporating such design modifications may enhance implant durability, reduce the risk of hardware failure, and improve the clinical outcomes of orthopedic surgeries including OWHTO by ensuring greater fixation stability throughout the bone healing process.

Our study has several limitations. First, this in vitro investigation focused exclusively on the head and neck junctions of screws without considering clinically relevant variables such as patient age, bone mineral density, or other physiological factors. Future study is needed to evaluate how the biomechanical advantages observed in this study translate into improved clinical outcomes. Second, although the reinforced screws demonstrated superior biomechanical performance, their increased stiffness may affect micro-motion at the osteotomy site was not evaluated. Micro-motion is an essential factor for bone healing in OWHTO, and its interaction with the reinforced screw design requires further investigation. Further investigations are warranted to assess how these structural modifications influence the bone-healing process in clinical applications. Finally, our study did not incorporate the manufacturer’s original design specifications for the screws. Instead, structural dimensions were assessed using direct caliper measurements. The absence of reference data from the original design specifications may introduce variability in interpreting the biomechanical impact of the structural modifications. Nevertheless, despite this limitation, the measurement methods employed in our study successfully identified structural differences in the head and neck junction between the two screw types. Furthermore, these structural differences were associated with significant biomechanical variations, underscoring the relevance and validity of our findings.

## Conclusions

Our study demonstrated that reinforcing the head and neck junctions of locking screws significantly improves their biomechanical performance, including compressive, fatigue, and torsional strengths. The reinforced screws showed greater resistance to mechanical failure and improved stiffness, suggesting their potential to reduce hardware failure risks in high-stress procedures such as OWHTO. These findings highlight the importance of structural modifications in enhancing implant durability and clinical outcomes in orthopedic surgeries.

## Data Availability

The data supporting the findings of this study are available from the corresponding author upon reasonable request for research purposes.

## Declarations

### Declarations

There is nothing to declare.

## Abbreviations

OWHTO: Opening Wedge High Tibial Osteotomy
LCP: Locking Compression Plate
ORIF: Open Reduction and Internal Fixation
MPa: Megapascal
Nm: Newton Meter
Hz: Hertz
SD: Standard Deviation
